# Sleep hygiene and sleep quality in Iranian adolescents during the COVID-19 pandemic

**DOI:** 10.1186/s40359-023-01165-8

**Published:** 2023-04-19

**Authors:** Azita Chehri, Maryam Shetabi, Habibolah Khazaie, Ali Zakiei

**Affiliations:** 1 The Center for Recent Findings in Applied Psychology, Kermanshah Branch, Islamic Azad University, Kermanshah, Iran; 2 Sleep Disorders Research Center, Kermanshah University of Medical Sciences, Kermanshah, Iran; 3 Sleep Disorders Research Center, Farabi Hospital, Kermanshah University Of Medical Sciences, Dovlat Abad Blvd, Kermanshah, Kermanshah Iran

**Keywords:** Sleep hygiene, Sleep quality, Adolescents, COVID-19

## Abstract

**Objectives:**

Sleep is one of the issues that attracted the attention of researchers during the COVID-19 pandemic. Researchers focused their studies on the prevalence of sleep disorders, sleep quality, and sleep duration. Sleep hygiene is a set of guidelines that play an important role in the sleep quality, the present study sought to evaluate the amount of sleep hygiene and sleep quality in Iranian adolescents and their relationship during the COVID-19 pandemic.

**Methods:**

The present study was conducted using a cross-sectional design. The research population consisted of all adolescents living in Kermanshah (western Iran) in 2021. The participants were a sample of 610 adolescents. They completed the Pittsburgh Sleep Quality Inventory and Adolescent Sleep Hygiene Scale.

**Results:**

The mean sleep quality score for the participants was 7.14 ± 2.47, indicating the high frequency of sleep problems affecting the participants. There were significant correlations between all components of sleep hygiene and sleep quality. There was also a significant correlation (r = − 0.46 between sleep hygiene and sleep quality (*p* < 0.001). No significant difference was observed in sleep hygiene and sleep quality between the male and female adolescents. The results showed that sleep hygiene subscales can predict sleep quality (R = 0.53, F = 39.20, *p* < .01).

**Conclusions:**

The data in this study confirmed the poor observance of sleep hygiene in adolescents during the COVID-19 pandemic and reported frequent sleep problems among the participants. The results also indicated a moderate relationship between sleep hygiene and sleep quality in adolescents. Thus, sleep hygiene components can be related to sleep quality.

## Introduction

The Covid-19 pandemic affected all people around the whole world and a large number of people lost their life due to the disease. The spread of this disease has forced people to stay at home and retreat into isolation. This social isolation and worries about the COVID-19 outbreak and its psychological consequences had adverse effects on people’s life [1]. Studies conducted in this field confirmed the prevalence of disorders such as anxiety, depression, and sleep problems [2]. These problems had many negative effects on adolescents. Disorders such as anxiety, depression, and stress were frequently reported in adolescents around the world [3]. Stressful life events, long-term confinement at home, sadness, domestic violence, and excessive use of the Internet and social media are factors that can affect the mental health of adolescents during this period [4]. Moreover, the closure of schools during the pandemic was also a problem that aggravated these consequences [5].

One of the issues that attracted the attention of researchers during the COVID-19 outbreak was the issue of sleep. Thus, researchers focused their studies on the prevalence of sleep disorders, sleep quality, and sleep duration [6–8]. Changes in educational programs and lifestyle changes were frequently reported during the pandemic, affecting the observance of sleep hygiene and, as a result, decreased sleep quality [8]. The importance of healthy sleep, especially at a young age, warrants further research in this field because healthy sleep is an essential part of normal growth and is especially important for health and developing learning abilities in school [9]. Besides, adequate sleep is vital for mental and physical health and plays an important role, especially in emotion regulation, cognition, psycho-social development, and physical development [10]. Healthy sleep also plays a vital role in public health [11]. On the other hand, inappropriate sleep patterns and sleep disorders cause a decrease in well-being, physical pain, a decrease in quality of life, and psychological disorders [12]. Sleep problems are associated with deliberate self-harm behaviors [10]. Furthermore, variables such as sleep quality and the severity of insomnia play a role in the occurrence of intentional non-suicidal self-injury (NSSI). Indeed, sleep problems can be considered a risk factor for NSSI [13]. In addition, decreased sleep quality can be associated with suicidal behaviors [14]. Accordingly, there is a need for further research on sleep quality, especially in critical situations [15].

Some have highlighted the significance of promoting and observing sleep hygiene as a protective factor contributing to preventing and managing infectious diseases [16]. The results of a study showed that the quality of sleep and sleep hygiene decreased during the COVID-19 pandemic, and emphasized that sleep hygiene is an issue that has been neglected during this period [17].

The role of gender in sleep-related research should be considered, the results of a study has shown that gender differences play a role in sleep quality and sleep hygiene, so that sleep quality is worse in girls, and the level of adherence to Sleep hygiene principles is less in girls [18], Therefore, in the present study, the role of this difference was also investigated.

Adolescents have an undeniable role in the future and progress of the community. Thus, paying attention to their issues and problems, especially sleep problems and sleep hygiene is a serious and important matter. Furthermore, exploring sleep quality in adolescents can contribute to the development of human knowledge in this field. Identifying the factors affecting sleep problems can help us find solutions to improve the quality of sleep. On the other hand, the COVID-19 pandemic and similar crises create stressful conditions for adolescents, which may adversely affect their sleep quality. Accordingly, the present study aimed to evaluate sleep problems and sleep hygiene in adolescents. In fact, this research sought to answer the following questions:

1. What was the state of sleep hygiene of Iranian adolescents during the corona pandemic period?
2. What was the state of sleep quality of Iranian adolescents during the corona pandemic period?
3. Is there a significant relationship between sleep hygiene and sleep quality in adolescents?
4. Can sleep hygiene predict sleep quality in adolescent?

## Methods

### Study design and participants

This study was conducted using a cross-sectional design. The research population consisted of all adolescents living in Kermanshah in 2021. According to the statistics released by the General Registry Office of Kermanshah Province, 421,266 adolescents were living in the province. The participants were selected from 11 to 18-year-old adolescents living in urban areas. In Morgan’s table, the maximum sample size for a population with more than 100,000 members is reported to be 384 persons. However, to ensure the credibility of the findings, 610 adolescents living in urban areas were selected as the participants in this study. The questionnaires were distributed online and in electronic form in cooperation with 7 psychological experts in schools from all areas of the city and among available adolescents. The questionnaires were completed after explaining the objectives of the study and assuring the participants about the confidentiality of their personal information. Additionally, they were assured that their information would remain confidential, and their informed consent was taken. The criteria for enrolling the adolescents in the study were being 11–18 years old, the willingness to participate in the study, and accuracy in answering the items in the questionnaires. The exclusion criteria were not having specified psychiatric diseases affecting sleep, substance abuse, and taking drugs affecting sleep. Since this study was conducted during the COVID-19 pandemic and given restricted access to the participants, the questionnaires were completed by the participants online through social media and networks. The protocol for this study was registered in the Sleep Disorders Research Center of Kermanshah University of Medical Sciences in Iran and was approved by the ethics committee of the university with the code IR.KUMS.REC.1401.009.

### Study instrument and variables assessment

#### The demographic questionnaire

The questionnaire assessed the participants’ demographic characteristics including age, education, gender, history of smoking and drug use, history of medical diseases, family status, and the number of hours of playing with electronic devices in 24 h (such as Xbox, PS4, computers, mobile phones, etc.)

#### Adolescent sleep hygiene scale (ASHS)

Adolescent Sleep Hygiene Scale is a 28-item self-report scale. The items are scored on a six-point Likert scale: Never (1), rarely (2), usually (3), sometimes (4), most of the time (5), and always (6). ASHS was developed by LeBourgeois et al. [19]. The Cronbach’s alpha for the whole scale was satisfactory, and its multidomain internal consistency, with Cronbach’s alpha ranging from 0.37 to 0.74, was lower than the recommended levels [19]. The scale has been validated in Iran by Chehri et al. They reported Cronbach’s alpha value of 0.71 to 0.75 for the scale and its reliability ranged from 0.82 to 0.87. A significant correlation was found between most of the revised Persian versions of the Adolescent Sleep Hygiene Scale (ASHS) and the Pittsburgh Sleep Quality Inventory. The results of this study indicated that the Persian version of the ASHS can be used as a reliable and valid tool to evaluate sleep hygiene practices among Persian-speaking adolescents [20]. A higher score on this scale indicates a higher level of sleep hygiene.

#### Pittsburgh sleep quality inventory (PSQI)

This 19-item instrument was developed by Buysse et al. [21] to measure sleep quality and help identify people who sleep well or badly. Cronbach’s alpha was 0.83 for the internal consistency of the inventory. The items in this self-report instrument are completed within 5 min. The psychometric properties of the inventory were assessed by Farahi et al. [21] and its Cronbach’s alpha was 0.77. The authors concluded that the psychometric properties of the Persian version of the PSQI are acceptable. Chehri et al. also validated the inventory by administering it to a sample of adolescents. Cronbach’s alpha was 0.72, showing that the Persian version of the inventory has acceptable psychometric properties [21]. A higher score on this inventory means more sleep problems. This questionnaire has 19 statements, and the answers to these items are on a four-point Likert scale from 0 to 3. A score of zero is given for the option of never, a score of one for the option of less than once a week, a score of two for the option of once or twice a week, and a score of 3 for the option of three or more times a week. And to evaluate the sleep quality, a score of zero is given for a very good option, a score of one for a relatively good option, a score of two for a relatively bad option, and a score of 3 for a very bad option.

### Data analysis

The collected data were analyzed using SPSS-22 software. Mean and standard deviation indices were used to describe the data. Pearson’s correlation test was performed to investigate the relationship between sleep hygiene and sleep quality. To interpret the correlation coefficients, we considered values below 0.40 as weak correlation, between 0.40 and 0.70 as moderate correlation, and above 0.70 as strong correlation [22]. Moreover, multivariate regression analysis was run to predict sleep quality based on sleep hygiene. The one-way analysis of variance (ANOVA) was also used to compare the mean scores among the groups. To check normality of the data, the Kolmogorov Smirnov test was used and the results showed a normal distribution. It should be mentioned that the significance level was considered *p* < 0.05 for all statistical tests.

## Results

The participants in this study were 610 adolescents including 336 females (55.1%) and 274 males (44.9%). The participants’ mean age was 15.01 ± 2.13 years. The results also showed that participants used electronic devices for 6.37 ± 5.01 h per day. Table 1 shows the descriptive statistics or various aspects of sleep problems by gender:

**Table 1:** Comparison of sleep problems in each domain among male and female groups

Domains	Total (M ± SD)	Male (M ± SD)	Female (M ± SD)	F	df	*p*-value	Effect size
Sleep latency	1.70 ± 1.01	1.57 ± 0.96	1.86 ± 1.05	11.94	1	0.001	0.019
Sleep disturbances	0.96 ± 0.50	0.96 ± 0.52	0.97 ± 0.45	0.061	1	0.803	0.001
Sleeping medication	0.22 ± 0.59	0.21 ± 0.79	0.24 ± 0.58	0.280	1	0.608	0.001
Daytime dysfunction	0.67 ± 0.79	0.69 ± 0.79	0.63 ± 0.80	1.06	1	0.303	0.002
Subjective sleep quality	1.03 ± 0.95	0.97 ± 0.92	1.10 ± 1	2.532	1	0.094	0.004
Sleep efficiency	2.55 ± 0.79	2.57 ± 0.78	2.54 ± 0.80	0.059	1	0.741	0.001
Sleep duration	5.44 ± 1.12	5.46 ± 1.13	5.42 ± 1.12	0.176	1	0.633	0.001
Sleep quality (total)	7.14 ± 2.47	6.98 ± 2.51	7.34 ± 2.40	3.125	1	0.078	0.005

*M* Mean, *SD* Standard deviation, *F* Fisher's F index value, *df* Degrees of freedom

As can be seen in the table above, the mean score for sleep problems reported by the participants through the Pittsburgh Sleep Quality Inventory was 7.14 ± 2.47. Besides, there was no statistically significant difference between the male and female participants. However, this value shows poor sleep quality among the adolescents in this study. A comparison of the sleep quality subscales indicated that, except for the sleep latency component, there was no significant difference between the male and female participants.

Table 2 shows the descriptive statistics for various subscales of sleep hygiene by gender:

**Table 2:** Comparison of sleep hygiene in each domain among male and female

Domains	Total (M ± SD)	Female (M ± SD)	Male (M ± SD)	F	df	*p*-value	Effect size
Physiological	22.06 ± 4.24	22.09 ± 4.24	22.03 ± 4.26	0.015	1	0.854	0.001
Behavioral arousal	12.27 ± 4.10	12.58 ± 4.08	11.89 ± 4.09	18.411	1	0.041	0.030
Cognitive/emotional	23.39 ± 6.34	23.39 ± 6.27	23.41 ± 6.43	0.002	1	0.971	0.001
Daytime sleep	9.09 ± 2.61	9.28 ± 2.56	8.86 ± 2.63	2.896	1	0.044	0.005
Sleep environment	24.36 ± 4.71	24.56 ± 4.77	24.10 ± 4.64	1.858	1	0.227	0.003
Sleep stability	10.72 ± 4.007	10.41 ± 3.83	11.10 ± 4.18	1.953	1	0.036	0.003
Sleep hygiene (total)	101.90 ± 17.04	102.32 ± 17.44	101.39 ± 16.54	0.452	1	0.502	0.001

As shown in Table 2, the mean score of sleep hygiene for the participants is 101.90 ± 17.04, indicating no significant difference between the male and female participants. However, a comparison of subscales of sleep hygiene suggested some differences between the male and female participants in terms of behavioral arousal, daytime sleep, and sleep stability, and the females obtained higher scores on these sleep hygiene subscales. By converting the scores of each participant to the mean, the ASHS mean score for all participants was 4.25 ± 0.71, indicating poor sleep hygiene among the participants. Table 3 shows an assessment of sleep hygiene in the participants.

**Table 3:** An assessment of sleep hygiene and sleep quality in the participants

	Variable	Frequency	Percent	Explain
Sleep quality	0–5	155	25.4	Good sleeper
	5–21	455	74.6	Poor sleeper
Sleep hygiene	< 3.8	160	26.2	Poor sleep hygiene
	3.8–4.9	325	53.3	Mediocre sleep hygiene
	> 4.9	125	20.5	Good sleep hygiene

According to the results in Table 3, 160 participants (26.2%) had poor sleep hygiene and 125 participants (20.5%) had good sleep hygiene. Data analysis also revealed that sleep quality was poor in 74.6% of the participants.

Table 4 presents the results of the Pearson’s correlation test for the relationship between sleep hygiene and sleep quality, and their subscales:

**Table 4:** correlation between sleep hygiene and sleep quality

Domains	r	*P*
Physiological	− 0.29	0.001
Behavioral arousal	− 0.31	0.001
Cognitive/emotional	− 0.38	0.001
Daytime sleep	− 0.42	0.001
Sleep environment	− 0.29	0.001
Sleep stability	− 0.13	0.001
Sleep hygiene (total)	− 0.46	0.001

*r* Pearson correlation

As can be seen, there were significant correlations between all subscales of sleep hygiene and sleep quality, most of these correlations are less than 0.40, which are weak correlations. Moreover, there was a significant correlation (r = − 0.46) between sleep hygiene and sleep quality (*p* < 0.001), It can be said that this correlation is moderate. In addition, there was a significant correlation (r = 0.12) between the hours of using electronic devices and sleep quality (*p* < 0.05), This is a weak correlation.

Multivariate regression analysis was run to predict sleep quality based on sleep hygiene subscales (Table 5). The results showed that sleep hygiene subscales can predict sleep quality (R = 0.53, R^2^ = 0.27, F = 39.20, *p* = 0.001). Accordingly, sleep hygiene subscales can explain 28% of sleep quality changes.

**Table 5:** Multivariate regression analysis for predicting sleep quality

Domains	Regression (R = 0.52, R^2^ = 0.27, F = 36.59)			
	B	β	t	*p*
Physiological	− 0.09	− 0.17	4.32	0.001
Behavioral arousal	− 0.09	− 0.16	4.008	0.001
Cognitive/emotional	− 0.09	− 0.22	5.34	0.001
Daytime sleep	− 0.20	− 0.28	4.89	0.001
Sleep environment	0.02	0.04	0.79	0.366
Sleep stability	0.009	0.05	0.43	0.201

*B* Unstandardized regression coefficient, *β* Standardized regression coefficient

## Discussion

The current study investigated sleep problems and sleep hygiene in Iranian adolescents during the COVID-19 pandemic. The results showed that the adolescents had poor sleep quality and hygiene during the COVID-19 pandemic. There was also a significant relationship between sleep hygiene and sleep quality, indicating that the greater the compliance with sleep hygiene, the fewer sleep problems and the higher the quality of sleep.

The results of a study showed an increase in sleep time and sleep latency, a change in waking time, poor sleep quality, and more frequent insomnia symptoms during the COVID-19 outbreak [23]. A meta-analysis study also showed that sleep disturbances were more frequent during the COVID-19 pandemic [24]. Other studies also reported similar results [25–28]. A systematic meta-analysis review of 250 studies on about half a million participants indicated that the estimated prevalence of sleep disorders (including poor sleep quality and insomnia), independent of any other variable, was 40% across all ages during the COVID-19 pandemic. The data also presented the same overall estimate of sleep disturbance, ensuring that this is likely a reasonable estimate of sleep disturbance associated with COVID-19, and reported that children and adolescents were the second most affected group with the overall prevalence rate of sleep disorders of about 46% [29]. The results of another study reported the prevalence of sleep disorders in children and adolescents during the COVID-19 pandemic to be 54% [30]. Thus, the findings of the present study confirming the high prevalence of sleep problems were in line with other studies in the literature.

The results of a study in Iran before the COVID-19 pandemic showed that a significant percentage of school-age children have sleep problems, including resistance to falling asleep and waking up during the night, difficulty waking up in the morning, insufficient sleep, and breathing problems in sleep. Moreover, these problems were more frequent in boys than in girls. The results also showed a significant relationship between sleep hygiene and sleep problems [9], as confirmed in the present study.

Sleep is a behavior that can be controlled by an individual during an epidemic outbreak. However, this is not a simple task because psychological worries and fear aggravate sleep problems [30]. New psychological concerns were reported by adolescents during the COVID-19 pandemic that may be related to sleep abnormalities [30]. Furthermore, anxiety, depression, irritability, impatience, inattention, and fear of COVID-19 were the predominant psychological problems during the COVID-19 pandemic [31] that could account for sleep problems.

The data in the present study showed that there is a relationship between non-observance of sleep hygiene and sleep problems, as reported in previous studies [32–34]. A review study showed that the components of sleep hygiene are associated with sleep quality [35]. An experimental study also showed a relationship between sleep hygiene and sleep quality in adolescents [36]. Sleep hygiene refers to routine practices that can facilitate and maintain quality sleep at night [37]. These practices include instructions for having a regular sleep and wake schedule, prohibiting activities that disrupt sleep, and managing thoughts and emotions before sleep. Hence, a person who adheres to these instructions can have a good sleep.

The findings of the present study also showed a relationship between the number of hours of using electronic devices and sleep quality among adolescents, indicating that the use of electronic devices such as mobile phones and tablets can be associated with sleep problems, as reported in previous studies [38–40].

Adolescence is a sensitive period and this period is associated with special psychological problems. On the other hand, cultural conditions have a great impact on psychological issues, considering that our study was conducted in Iran and this country has different cultural conditions from Western countries. We must keep these conditions in mind when explaining the results. Among these conditions is the issue of individual independence, which is not much considered by families in Iranian culture.

### Limitation of the study

The sampling method in our research was not random and this is one of the limitations of the study. Also, the sample included urban adolescents and people living in rural areas were not included in the study. In fact, this study was conducted on a sample of Iranian adolescents living in urban areas. Thus, caution should be taken to generalize the results to other societies. Furthermore, this study employed a correlational design, so it was not possible to infer cause-effect relationships between the variables. This study was also conducted using cross-sectional data, while sleep hygiene and sleep quality and its consequences need longitudinal studies to confirm the cause-and-effect relationship during the COVID-19 pandemic.

This research project was conducted on a large sample during the COVID-19 pandemic, which created stressful conditions for people in the community. Thus, the findings of this study can be of interest to therapists and planners.

## Conclusion

The findings of the present study confirmed poor sleep hygiene and frequent sleep problems among adolescents during the COVID-19 pandemic. The results also showed a significant relationship between the observance of sleep hygiene and sleep quality in adolescents, and sleep hygiene components can predict sleep quality.

The findings of this study can have some implications for families and parents. Poor sleep quality and poor sleep hygiene in adolescents contribute to countless problems. Thus, parents need to receive adequate training on adolescent sleep hygiene. Moreover, counseling and psychotherapy centers can organize workshops and seminars on sleep hygiene and sleep quality and provide some instructions for adolescents to improve their sleep quality and hygiene.

Future researches should evaluated other components related to sleep quality in adolescents, it is also necessary to conduct all-round investigations (including psychological and sociological) in the field of sleep quality in adolescents.

## Data Availability

The datasets used during the current study are available from the corresponding author on reasonable request.
